# Toxicogenic Fungi, Aflatoxins, and Antimicrobial Activities Associated with Some Spices and Herbs from Three Selected Markets in Ho Municipality, Ghana

**DOI:** 10.1155/2022/7195890

**Published:** 2022-06-24

**Authors:** Nii Korley Kortei, Barnabas Teye Djaba, Clement Okraku Tettey, Alfred Ofori Agyemang, Enoch Aninagyei, Edward Ken Essuman, Adjoa Agyemang Boakye, Theophilus Annan

**Affiliations:** ^1^Department of Nutrition and Dietetics, School of Allied Health Sciences, University of Health and Allied Sciences, PMB 31, Ho, Ghana; ^2^Department of Biomedical Sciences, School of Basic and Biomedical Sciences, University of Health and Allied Sciences, PMB 31, Ho, Ghana; ^3^Institute of Traditional and Alternative Medicine, University of Health and Allied Sciences, PMB 31, Ho, Ghana; ^4^Food Microbiology Division, Council for Scientific and Industrial Research-Food Research Institute, P.O. Box M20, Accra, Ghana

## Abstract

Spices and herbs are widely used food ingredients that enhance most organoleptic features of prepared foods. They are also used for medicinal and preservative purposes. Spices and herbs are potential carriers of bacteria, yeasts, and molds due to the nature of cultivation, harvest methods, storage conditions, packaging procedures, distribution, sale, and general handling. Although some fungi have been identified to be associated with most spices and herbs elsewhere in the world, little has been done on the presence of fungi in spices and herbs in Ghana. This study sought to identify the toxicogenic fungal profiles, mycotoxins (aflatoxins) present in some herbs, bay leaf (*Laurus nobilis*) and garden egg leaves (“gboma”) (*Solanum macrocarpon*), and spices, ginger (*Zingiber officinale*) and “dawadawa”(*Parkia biglobosa*), as well as to investigate the antimicrobial properties of the selected herbs and spices. The decimal reduction technique was used to plate onto Dichloran Rose Bengal Chloramphenicol (DRBC) agar media plates for fungal growth. Aflatoxin detection was carried out with high-performance liquid chromatographer connected to a fluorescence detector (HPLC-FLD). Antimicrobial properties were carried out using the agar diffusion method on solidified, freshly prepared Mueller-Hinton agar. A total of 12 species belonging to 7 genera, *Aspergillus* (*niger*, *flavus*, *fumigatus*, and *ochraceus*), *Fusarium* (*oxysporum*, *verticillioides*), *Mucor* (*racemosus*), *Penicillium* (*digitatum*, *expansum*), *Rhizopus* (*stolonifer*), *Rhodotorula* sp., and *Trichoderma harzianum*, were identified as fungal contaminants. *Fusarium oxysporum* was the most predominant species identified. Fresh ginger recorded the greatest number of colony-forming units (3.71 log_10_ CFU/g) with bay leaves recording the least number of colony counts (2.36 log_10_ CFU/g). Mycotoxin concentration detected in gboma was2.06 ± 0.07 *μ*g/kgand in dawadawa was2.13 ± 0.09 *μ*g/kg; however, mycotoxins were not detected in bay leaf and ginger. Ginger exhibited antibacterial activity against all bacteria ranging from 7.0 ± 0.0 mm to 12.0 ± 5.66 mm zones of inhibition. Ginger, bay leaf, and gboma extracts displayed fair antimicrobial activity against the bacteria investigated. On the other hand, *dawadawa* generally produced the least resistance against the five bacterial species but exhibited the highest zone of inhibition. All samples were slightly acidic with pH readings ranging from 5.81 to 6.76.

## 1. Introduction

Spices and herbs are products of plant origin which are used as ingredients in foods to perform several organoleptic functions, taste, flavor, smell, color, etc., as well as dietetic functions [1]. Spices have been known and used for years among all regions and groups of people in the world [2]. They are cultivated, harvested, and sold out in very large to small quantities to the general population. Most spices and herbs have great nutritional properties; they have been identified as natural antioxidant sources and hence play a crucial role in the chemoprevention of illnesses caused by lipid peroxidation [3]. Most of these spices are rich in phytochemicals [3].

Spices and herbs can be contaminated with toxigenic fungi and may lead to the production of mycotoxins [4]. Sufficient evidence has proven that spices are commonly heavily contaminated with xerophilic storage molds and bacteria [5, 6]. Additionally, inappropriate handling and other unfavourable conditions after harvesting, storage, processing, packaging, and distribution could leave these food products with traces of fungi on them [5].

In the Ghanaian market, there are numerous mixtures of spices that are well patronized. Common spices such as onion, pepper, ginger, nutmeg, rosemary, and garlic are used in the preparation of household cookery and foods sold at restaurants and food joints or chop bars (local eateries) [7]. Nutmeg and aniseed cloves are ocimum, which are most frequently used in the food industry as food additives for flavoring and health effects [8], while dawadawa is widely used in the northern parts but scarcely used domestically in the southern parts of Ghana. Spices and their extracts, particularly their oils, are receiving attention and are being widely studied as potential antimicrobial agents, which may be important for food preservation and the control of human diseases of microbial origin. Previous work done by some researchers in Ghana reported these local spices to have several activities against microbial infections [7, 9–11]. This may probably be due to the bioactive phytochemicals such as terpenoids, flavonoids, phenolics, and alkaloids present in these herbs and spices, which makes it possible to reduce pathogen populations and curb mycotoxin proliferations in foods.

Some fungi produce secondary metabolites known as mycotoxins, which are capable of causing several diseases in humans and some animals as well. Mycotoxins are inevitable contaminants found in food grains, feeds, medicinal herbs, and spices, and they pose a health risk to both animals and people [12]. As spices and herbs are widely used for various needs by Ghanaians, it has become important to determine the possible species of fungi that are associated with different local spices. Spices and herbs are gaining importance in recent years as potential sources of natural food preservatives due to the growing interest in the development of safe and effective natural food preservation [13]. Efforts are being made by food scientists to gradually reduce the use of artificial synthetic preservatives in the food industry because they are known to cause some adverse health effects, especially cancer. A thorough look must be made into ensuring the maximum safety of food products that are preserved by these natural preservatives: spices, herbs, and their extracts.

This study is aimed at updating the fungal profile, mycotoxins, and antimicrobial properties of spices and herbs from three selected markets in the Ho municipality, Ghana.

## 2. Materials and Methods

### 2.1. Study Design

A quantitative experimental study design was employed in this study.

### 2.2. Study Site

Samples were obtained from three selected markets in the Ho municipality, namely, Ho main market (1), Ahoe satellite market (2), and Dome market (3). All other procedures were carried out in the University of Health and Allied Sciences (UHAS) food laboratory located on the Dave campus.

### 2.3. Sampling and Sample Preparation

Samples of spices and herbs are, namely, bay leaf (BL) (fresh), ginger (fresh ginger = GF, powdered ginger = GP), dawadawa (DAW), and gboma (GB) (fresh) (Table 1). A sample size of forty-five (45) individual units was used. Five different spice and herb samples were purchased at three points per each of the three different markets. The samples were randomly sampled over a two-week period in July and August 2020. Approximately 100 g of each sample was collected and stored in sterile specimen containers (Nasco, USA) and transported in an ice chest freezer (Thermos 7750, China) with cold packs at a temperature of 10°C under aseptic conditions to the UHAS laboratory for microbiological analysis within 2 hours of collection [14].

Differences in moisture contents of the samples were corrected by using the formula

(1) $$\begin{array}{c} C_{\text{dry}}=\frac{C_{\text{wet}}}{\left(1-\%\text{Moisture}\right)}. \end{array}$$

#### 2.3.1. Fungal Plating

One gram (1 g) of each sample was transferred into 9 ml of distilled water. The samples were then vortexed for 1 minute at a moderate speed. One milliliter of each sample was plated onto the media plates and incubated at 25°C for 7 days. All samples were weighed using an electronic balance (OHAUS®) with a readability of 0.01 g. Each stock solution was serially diluted in 9 ml of peptone (0.1%) water in tenfold increments up to 10^−3^. One milliliter (1 ml) of each serial dilution was plated onto Dichloran Rose Bengal Chloramphenicol (DRBC) (Oxoid CM727, United Kingdom). Agar media plates were prepared according to the manufacturer's instructions and incubated at 25°C for 5-7 days [15].

#### 2.3.2. Fungal Enumeration and Identification

Enumeration was carried out by a colony counter. Fungal counts were recorded in standard form and later transformed into a logarithmic scale. Colony-forming unit (CFU) per gram was calculated using the formula [14, 16]

(2) $$\begin{array}{c} \frac{\text{CFU}}{g}=\frac{\left(\text{no}.\text{of colonies}\times \text{reciprocal of the dilution factor}\right)}{\text{volume of culture plate}}. \end{array}$$

Percentage occurrence of fungal species was calculated using the formula

(3) $$\begin{array}{c} \text{Percentage }\left(\%\right)\text{ occurrence of fungal species}=\frac{\text{Number of fungal species}}{\text{Total number of fungi isolated}}\times 100. \end{array}$$

#### 2.3.3. Lactophenol Cotton Blue Tease Procedure

A drop of Lactophenol Cotton Blue (LPCB) dye was placed on the slide, and a sterile iron needle was used to transfer a tiny piece of a colony of Lactophenol Cotton Blue Dye on the slide. The colony was then teased into minuscule pieces using an iron needle. The slide was covered with a coverslip.

**Figure 1:**
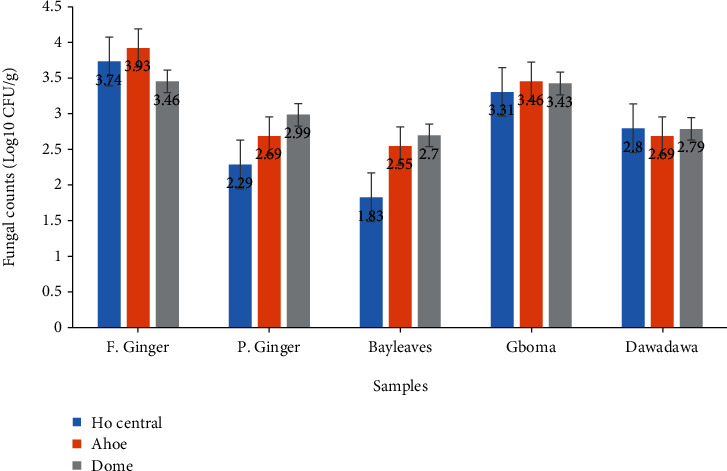
Mean fungal counts on samples from the three markets. Data presented are means ± standard error.

**Figure 2:**
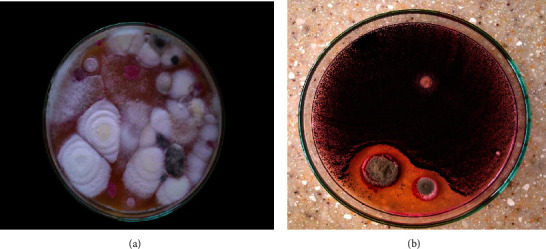
Macroscopic view of fungal species: (a) *Fusarium oxysporum* on gboma and (b) *Aspergillus niger* in dawadawa on DRBC agar.

**Figure 3:**
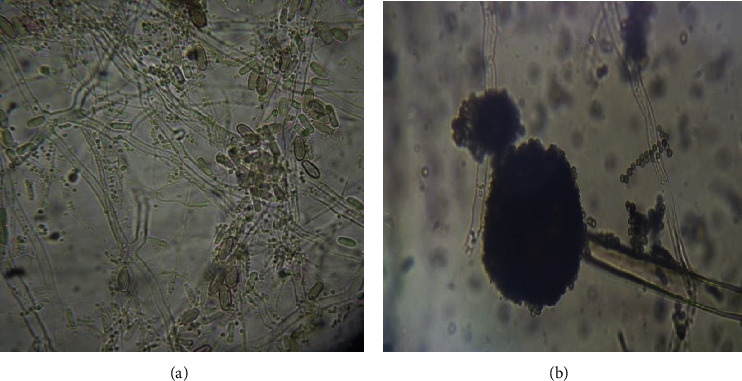
Microscopic views of (a) *Fusarium oxysporum* (×400) and (b) *Aspergillus niger* (×400).

Identification of fungi was done macroscopically (texture and color of the plate) and microscopically by observation of morphological features under the microscope (Figures 1–3).

### 2.4. Identification

Molds that appeared were identified by their culture and morphological characteristics (Figures 2 and 3) using standard identification manuals [17, 18].

### 2.5. Aflatoxin Analysis

#### 2.5.1. Extraction of Samples

The European Committee for Standardization (CEN) official method EN14123 [19] was used to extract and quantify AFB_1_, AFB_2_, AFG_1_, and AFG_2_ from the samples. Methanol in water (200 ml) (8 + 2) and 5 g NaCl were used to extract 20 g of sample. Fat samples containing more than 50% fat were extracted with 100 ml of hexane in the normal methanol extraction solvent. One hundred milliliters (100 ml) of hexane was added to 200 ml of methanol. After homogenization, a separation funnel was used to separate the hexane, which became the upper layer. The mixture was homogenized for 3 min at 3000 rpm (2 min) and 3500 rpm (1 min). The extracts were filtered and 10 ml of the filtrate added to 60 ml of phosphate buffer saline (PBS) for solid-phase extraction using a preconditioned immune affinity column specific for AFB_1_, AFB_2_, AFG_1_, and AFG_2_. The 70 ml filtrate-PBS mixture was loaded onto the preconditioned column and allowed to elute by gravity at a flow rate of 1 ml min^−1^. This was followed by a cleanup with 15 ml distilled water at a flow rate of 5 ml min^−1^. Aflatoxins were eluted in two steps into a 5 ml volumetric flask with 0.5 ml followed by 0.75 ml of methanol (HPLC grade) and allowed to elute by gravity. Deionized water was used to make up the volume of eluate to 5 ml and eluate vortexed and 2 ml pipetted into HPLC vials for quantification.

#### 2.5.2. HPLC Parameters

Injection volume: 10 *μ*l, flow rate: 1 ml/min, column temperature: 35°C, excitation wavelength: 360 nm, emission wavelength: 440 nm, mobile phase composition: water/acetonitrile/MeOH (65 : 15 : 20 *v*/*v*/*v*), postcolumn derivatization: Kobra cells. HPLC column specification Spherisorb ODS1-Excel (4.6 mm × 25 cm), 5 *μ*m particle size, 250A pore size.

#### 2.5.3. Limit of Detection/Quantification (LOD/LOQ)

The limit of detection and quantification (LOD/LOQ) of the HPLC was estimated by making a calibration curve around the standard used for spiking 5 *μ*g/kg (the lowest concentration range of the calibration curve). Blank did not produce any signal, so LOD and LOQ were calculated as

(4) $$\begin{array}{c} \text{LOD}=3\times \frac{\text{standard deviation}}{\text{slope}},\\ \text{LOQ}=3\times \text{LOD}. \end{array}$$

#### 2.5.4. Measurement Accuracy

Spiking of pure aflatoxin standard solution was done to ensure the measurement accuracy of the analysis. Three levels of spiking were done at the lower, middle, and upper concentration range of the calibration curve concentrations (5 *μ*g/kg, 15 *μ*g/kg, and 30 *μ*g/kg). Spike volumes of pure standards were calculated as

(5) $$\begin{array}{c} \frac{\left[\text{Sample weight }\left(\text{g}\right)\times \text{spike concentration }\left(\text{ppb}\right)\right]}{\left[\text{Concentration of standard }\left(\mu \text{g}/\text{ml}\right)\right]}. \end{array}$$

Spike volumes were distributed evenly on aflatoxin-free sample (blank), and the spiked sample was analyzed for percentage recovery which was calculated as

(6) $$\begin{array}{c} \left[\frac{\left(\text{Concentration measured in spike}–\text{concentration measured in blank}\right)}{\left(\text{spiked amount}\right)}\right]\times 100. \end{array}$$

#### 2.5.5. Measurement Precision

Repeatability and intermediate precision analyses of an Internal Reference Material (IRM) were used to ensure the measurement precision of the method. For repeatability analysis, 10 parallel extractions of the IRM were done by the same analyst at the same time using the same HPLC, and the relative standard deviation between the results was calculated. For intermediate precision, 10 extractions of the IRM were done on different days by different analysts, and the relative standard deviation between the results was calculated according to Kortei et al. [20]. The relative standard deviations were calculated as

(7) $$\begin{array}{c} \left[\frac{\text{Standard deviation}}{\text{mean}}\right]\times 100. \end{array}$$

### 2.6. Antimicrobial Sensitivity

#### 2.6.1. Plant Extraction for Antimicrobial Sensitivity Test

All samples (ginger, bay leaf, gboma, and dawadawa) were pulverized after which an initial mass (100 g) was weighed into clean conical flasks. About 200 ml of methanol was added to each sample of powdered spice in conical flasks. The conical flasks were covered with aluminium foil to prevent evaporation and kept for 48 hours at 28°C away from light and subjected to occasional shaking/stirring to enhance the extraction. The resulting solution was filtered using filter paper, and the filtrates were evaporated to dryness in a Heratherm oven at a temperature of 40°C for 48 hours. The dried weights of the extracts were determined and stored in falcon tubes. Plant extracts in falcon tubes were kept in a refrigerator at 8°C for further analysis.

#### 2.6.2. Determining the Antimicrobial Activity of Samples

Using the agar diffusion method, the extracts were concentrated and tested for antibacterial activity against *Pseudomonas aeruginosa*, *Klebsiella pneumoniae*, *Staphylococcus aureus*, *Salmonella typhi*, and *Streptococcus mutans* obtained from the Microbiology Unit, Department of Biomedical Sciences, University of Health and Allied Sciences. The agar diffusion method as previously used by Perez [21] and modified by Ahmad and Beg [22] was used. The test organisms were subcultured on Mueller-Hinton agar at 37°C between 18 and 24 hours. An inoculum (500 *μ*l) was adjusted to 1.5 × 10^8^ CFU/ml using 0.5 McFarland standard. On each Mueller-Hinton agar plate, each test organism was evenly spread using a sterile microbiological loop. The plate was incubated at room temperature (approx. 25°C) for 10 minutes. Subsequently, seven wells (diameter: 8 mm) were aseptically created into the agar medium. Four concentrations of each sample (500 mg/ml, 250 mg/ml, 125 mg/ml, and 62.5 mg/ml) of 70% methanol extraction were prepared. Into each of the wells, 100 *μ*l (5 mg/ml) spice and herb extracts was aseptically dispensed into six individual wells while 30 *μ*l of a reference drug (chloramphenicol (CPL) (concentration 5 mg/ml)) was dispensed aseptically into the seventh well. The Mueller-Hinton agar plates were incubated aerobically between 18 and 24 hours. The zones of inhibition of the plant extracts were measured and compared with those of the reference drug.

### 2.7. Ethical Considerations

Ethical clearance was sought from the University of Health and Allied Sciences Research and Ethics Committee (UHAS-REC) prior to the conduct of the research (protocol ID UHAS-REC A.9 [47] 20-21). All COVID-19 safety protocols were observed throughout the study.

### 2.8. Statistical Analysis

Linear regression analysis was used to calculate aflatoxin concentrations. Fungal counts were done in standard form but were transformed into logarithm. Antimicrobial activity results were presented as means and standard deviation. One-way ANOVA was performed, and means were separated using Duncan's Multiple Range Test (DMRT). A *p* value less than 0.05 was considered significant. Analyses were done using SPSS version 23 (USA, Virginia).

## 3. Results

### 3.1. Fungal Counts

There was an average fungal count ranging from 3.46 log_10_ CFU/g to 3.93 log_10_ CFU/g for fresh ginger samples. Fungal counts of fresh ginger samples from Ahoe and Ho central were comparable (*p* > 0.05). Similarly, counts from Ahoe and Dome were not comparable (*p* > 0.05). However, samples from Ho central and Dome were not significantly different (*p* < 0.05). There was observed comparatively higher counts for fresh ginger samples obtained from all three markets (Ho central, Ahoe, and Dome) than any other spice or herb (Figure 1).

The mean fungal counts obtained on DRBC agar media for powdered ginger ranged from a minimum value of 2.29 log_10_ CFU/g to a maximum of 2.99 log_10_ CFU/g (Figure 1). Ahoe and Dome showed no significant difference (*p* > 0.05). Ho central and Dome were comparable (*p* > 0.05).

For bay leaf samples, there was a mean fungal count range of 1.83 log_10_ CFU/g to 2.70 log_10_ CFU/g. Gboma samples from the three markets had a mean fungal count range of 3.31 log_10_ CFU/g to 3.46 log_10_ CFU/g. Counts from Ho central market were significantly (*p* < 0.05) lower than the other two markets.

There was no statistically significant difference (*p* > 0.05) in fungal counts among the three markets (Ho Central, Ahoe, and Dome) for all spices of gboma and dawadawa. For dawadawa samples, there was a mean fungal count ranging from 2.69 log_10_ CFU/g to 2.81 log_10_ CFU/g.

### 3.2. Percentage Occurrence of Fungal Species

A pooled data of fungi in herbs and spices from all three markets (Ho central, Ahoe, and Dome) comprising a total of twelve (12) fungal species were isolated and identified. These belonged to seven (7) genera: *Aspergillus*, *Fusarium*, *Mucor*, *Penicillium*, *Rhizopus*, *Rhodotorula*, and *Trichoderma*. The species were *Aspergillus niger*, *A. flavus*, *A*. *fumigatus*, *A. ochraceus*, *Fusarium oxysporum*, *F. verticillioides*, *Mucor racemosus*, *Penicillium digitatum*, *P. expansum*, *Rhizopus stolonifer*, *Rhodotorula species*, and *Trichoderma harzianum* (Tables 2 and 3). All species identified were molds with the exception of *Rhodotorula* species which was the only yeast among the fungi.

For the powdered ginger sample, four fungal species were identified, namely, *A. niger* (18.3%), *A. fumigatus* (23.3%), *A. ochraceus* (5.2%), and *Fusarium oxysporum* (53.2%). A total of nine fungal species were identified on fresh ginger samples: *A. niger* (7.3%), *A. flavus* (9.1%), *A. fumigatus* (5.7%), *F. oxysporum* (8.4%), *F. verticillioides* (27.7%), *Penicillium digitatum* (9.3%), *Rhizopus stolonifer* (21.8%), *Rhodotorula* spp. (7.0%), and *Trichoderma harzianum* (3.7%). A total of nine fungal species, namely, *A. niger* (22.3%), *Fusarium oxysporum* (20.3%), *A. fumigatus* (5.0%), *Penicillium digitatum* (5.0%), *A. flavus* (6.7%), *Trichoderma harzianum* (1.0%), *Mucor racemosus* (17.0%), *A. ochraceus* (2.7%), and *Rhizopus stolonifer* (20.0%), were isolated for bay leaf samples. Gboma samples had a total number of eight fungal species identified: *A. niger* (30.0%), *A. flavus* (4.3%), *A. fumigatus* (9.7%), *F. oxysporum* (16.6%), *F. verticillioides* (9.0%), *Penicillium expansum* (24.4%), *Rhodotorula* spp. (1.4%), and *Trichoderma harzianum* (3.7%), and in dawadawa samples, nine species of fungi were identified. These were *A. niger* (3.5%), *A. fumigatus* (3.6%), *A. ochraceus* (3.0%), *Fusarium oxysporum* (19.3%), *Mucor racemosus* (1.1%), *Penicillium digitatum* (18.4%), *R. stolonifer* (34.4%), *Rhodotorula* spp. (1.3%), and *Trichoderma harzianum* (15.3%) (Table 2).

*Fusarium oxysporum* had the highest percentage occurrence in all samples (23.54%) (Figures 2 and 3) compared to the other species identified, occurring at least once in each of the samples tested. On the other hand, *Rhodotorula* sp. occurred the least in all samples compared to the other species identified.

### 3.3. Antibacterial Activities of Samples

**Figure 4:**
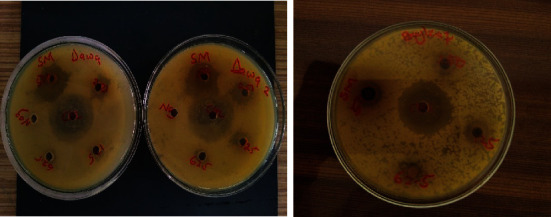
Zone of inhibition exhibited by (a) dawadawa and (b) bay leaf extracts against *Streptococcus mutans*.

The antibacterial activity of extracts from the various samples is presented in Table 4. Methanolic extracts of the four spices and herbs as tested against five bacterial species produced the following results: ginger extract showed inhibitory zones ranging from 4.5 ± 6.36 mm to 9.5 ± 0.70 mm against *Pseudomonas aeruginosa* and 10.5 ± 3.54 mm to 12.0 ± 5.66 mm. There was no significant (*p* = 0.16) difference observed. Bay leaf extract exhibited growth inhibition against *Pseudomonas aeruginosa* (6 ± 0.00 mm for 62.5 mg/ml to 9 ± 0.00 mm at 500 mg/ml) (Figure 4) and showed no significant (*p* = 0.10) difference. Ginger methanolic extract was potent against all bacteria investigated (Table 4). There was no zone of inhibition for all concentrations of bay leaf extract as tested against *Klebsiella pneumoniae* and *Salmonella typhi* (Table 4).

At 500 mg/ml, gboma extract exhibited an inhibitory zone of 13 ± 1.41 mm and 7.5 ± 0.71 mm at 62.5 mg/ml against *Pseudomonas aeruginosa* and there was a range of no zone of inhibition.

Gboma extract performed fairly against *Pseudomonas aeruginosa* with a range of 7 ± 0.00 mm to 13 ± 1.41 mm zone of inhibition where there was a statistically significant difference among the means of the various concentrations (*p* = 0.008). Extracts from the four samples showed poor antibacterial susceptibility to *Klebsiella pneumoniae*; gboma extract inhibited the growth of *Klebsiella pneumoniae* at 500 mg/ml (9.5 ± 0.71 mm), 250 mg/ml (8 ± 1.41 mm), and 125 mg/ml (8 ± 0.00 mm); there was a statistically significant difference (*p* < 0.001) among mean zone of inhibition for the various concentrations of gboma extracts. For *Salmonella typhi*, only ginger extract produced zones of inhibition which ranged from 7 ± 0.00 mm to 11 ± 2.83 mm diameter. There was a statistically significant difference (*p* = 0.001) between the zones of inhibition for the various concentrations.

### 3.4. Mean Aflatoxin Counts

**Figure 5:**
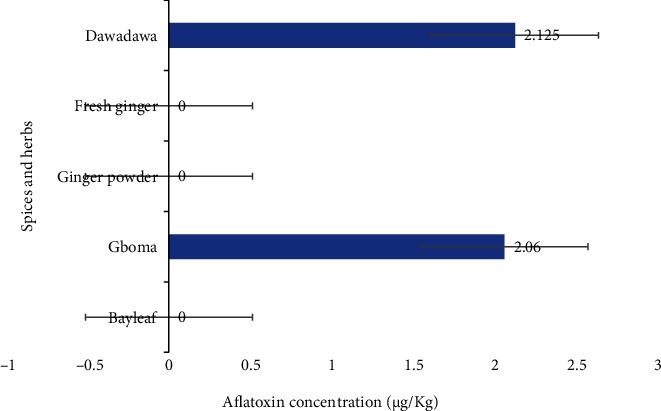
Mean aflatoxin concentrations in spices and herbs of samples from the Ho municipality.

Aflatoxin counts investigated in the various spice and herb samples are represented in Figure 5. Bay leaf and ginger (fresh and powdered) samples had no aflatoxins detected in them. Gboma samples tested recorded a mean aflatoxin count of 2.06 ± 0.50 *μ*g/kg, whereas dawadawa had a mean aflatoxin concentration of 2.13 ± 0.61 *μ*g/kg (Figure 5).

### 3.5. pH of Samples

The pH readings of the four spice and herb samples are presented in Table 5. Values of 6.56, 5.81, 6.20, and 6.76 were recorded for ginger, gboma, bay leaf, and dawadawa, respectively. The pH value for gboma (5.81) was significantly lower (*p* < 0.05) than that for the other samples.

## 4. Discussion

### 4.1. Fungal Counts

The count of the fungal species in the spices and herbs that were studied which ranged from 3.71 log_10_ CFU/g (5129) in fresh ginger to 1.83 log_10_ CFU/g in bay leaf is comparable to fungal counts of common spices studied in Saudi Arabia market (2325–6800 CFU/g) with the highest fungal counts also reported in ginger samples [23]. Similar studies reported 1120-1580 CFU/g (log_10_ 3.049-3.198 CFU/g) in some herbs and spices in Bahrain [24] and 1 × 10^3^–6 × 10^3^ CFU/g (log_10_ 3.0-3.778 CFU/g) in herbs and medicinal plants in Iraq [25]. However, other studies have reported much lower resident mycoflora ranged between 1.22 and 1.88 log_10_ CFU/g samples in some spices in Ghana [9, 26, 27]. Variation between fungal counts is to be expected because of sample and sampling variability, country of origin, and differences in processing and storage practices. For example, all spices in the present study manifest comparable species percentage occurrence and fungal counts to those reported by Chourasia [28]. The source of these foodstuff contaminants may be the result of natural extraneous pollution with dust particles containing spores from the field or storage [24, 29–31].

The observed higher fungal counts in ginger could be attributed to the soil origin of the rhizome, which is known to be a principal reservoir of all microorganisms. Zhang et al. [32] highlighted that variation in fungal counts could also be attributed to differences in compliance with good agricultural practices (GAP) as well as good manufacturing practice (GMP) conditions during the growing, processing, or storage of the raw material. Furthermore, the effect of storage on the viability of fungal propagules could also be worth considering.

Interestingly, another research work in Qatar found no fungal contamination in ginger, curry, and garlic samples but high fungal inoculum in chili powder [33]. Comparing fresh ginger with powdered ginger samples, fungal counts were higher in fresh ginger.

### 4.2. Fungal Species

Twelve distinct fungal species belonging to six genera were isolated from the four spices tested. *Aspergillus* species were the most isolated genus, and *Fusarium oxysporum* was the most isolated fungal species identified as it occurred in eleven out of a total of fifteen individual samples investigated. Fungal species occurrence in this study was sporadic with respect to the spice and herb. Ahene et al. [9] isolated similar species of fungi from raw spices and the spice products which belonged to eight (8) genera, *Absidia*, *Aspergillus*, *Cladosporium*, *Fusarium*, *Rhizopus*, *Penicillium*, *Neurospora*, *Eurotium*, but *Aspergillus species* (*A. flavus*, *A. fumigatus*, *A. alutaceus*, *A. niger*, and *A. sulphureus*) were the predominant species isolated. Mandeel [24] isolated some fungal species, and the most predominant fungal genera encountered were *Aspergillus*, *Penicillium*, *Rhizopus*, *Cladosporium*, and *Trichoderma*. In their work, yeasts were also frequently recovered, but not identified. Painstil [27] examined mycoflora in stored fresh ginger and powdered ginger. They recorded the occurrence of *Aspergillus flavus*, *Aspergillus fumigatus*, *Aspergillus nidulans*, *Aspergillus ochraceus*, *Aspergillus glaucus*, *Aspergillus niger*, *Cladosporium herbarum*, *Fusarium*, *Penicillium digitatum*, and *Trichoderma viride*. These fungal species showed five patterns of infection. In Indonesia, Nurtjahja et al. [34] reported that all tested spices were contaminated by storage fungi of which species of *Aspergillus* were the most commonly isolated molds followed by species of *Fusarium*, *Mucor*, *Penicillium*, and *Rhizopus*. The greatest number of *Aspergillus flavus* isolates was found on white pepper, followed by nutmeg, cardamom, and black pepper.

Toma and Abdulla [25] recorded ten different fungal genera, and 16 species were isolated and identified as *Alternaria alternata*, *Aspergillus* spp., *Gliocladium* sp., *Hyalodendron diddeus*, *Memnoniella* sp., *Penicillium* spp., *Rhizopus* spp., *Syncephalastrum* sp., *Cladosporium lignicolum*, and *Ulocladium botrytis* in spices and herbal medicinal plants in Iraq. Hashem and Alamri [23] reported *Aspergillus*, *Penicillium*, and *Rhizopus* as the most predominant fungal genera encountered in their research on spices. Samples obtained from sumac encountered very unusual colony counts indicating its antifungal affluences. *Alternaria* was characterized by *Alternaria alternate*. Ath-Har et al. [35] reported that *A. flavus*, *A. niger*, *Aspergillus nidulans*, *A. sydowii*, *A. ochraceus*, *Penicillium*, and *Rhizopus* spp. were most frequently isolated from spices and drug plants. Bugno et al. [36] showed that the predominant mycoflora obtained was distributed in 10 genera. The genus *Aspergillus* was the most dominant genus recovered (179 isolates) followed by *Penicillium* (44 isolates). The presence of a wide range of storage fungi indicates that considerable improvements could be made during postharvest storage. The dominance of *Aspergillus* and *Penicillium* spp. in all examined medicinal plant samples and spices was in accord with the results of Takatori et al. [37], Aziz et al. [29], and Hashem and Alamri [23], who stated that *Aspergillus* and *Penicillium* spp. were the main components of cardamom, cinnamon, fennel, coriander, cumin, black cumin, and white pepper, all of which are common in the food industry. They found a high degree of contamination in all samples.

Fresh ginger also outweighed powdered ginger samples for the occurrence of fungal species. Nine individual fungal species were isolated from fresh ginger, whereas four fungal species were isolated from powdered ginger samples. This is because moisture facilitates the growth of fungi [38]; powdered ginger has a low moisture content as is not with fresh ginger. Bay leaf samples had the lowest plate counts compared to the other three spices and herbs (Figure 1). This is attributed to the fact that the bay leaf samples were dry and thus had low moisture content, thereby reducing the chances of survival of microorganisms (fungi) on the herb spice. This notwithstanding, there was a total number of nine fungal species isolated from bay leaf samples tested with low percentage occurrence levels (Figure 2).

Presently, the prime challenge of toxicogenic fungi and mycotoxin proliferation in our foods is the link with climate change. There is now a widespread consensus that the world is warming at an unparalleled rate, and this is expected to seriously affect our crop production as well as the phyllosphere microflora of these crops. The irrepressible growth of *A. flavus* under extreme heat and dry conditions is an expected and emerging dilemma mainly in many parts of the world (Serbia, Hungary, etc.) where there were very low reports of mycotoxin contamination [39]. However, surges in maize contamination were observed after prolonged hot and dry weather. Due to this, the world's largest agricultural food exporters such as Brazil, Argentina, and some parts of Asia including China and India have been identified as hot spots for the impacts of climate change. From a food security viewpoint, a more precise forecast of the impacts of climate change on mycotoxins needs to be addressed to prevent conceded food sustainability, which possibly results in negative social consequences.

### 4.3. Aflatoxin Contamination

Aflatoxin concentrations recorded in this present work were 2.06 ± 0.50 *μ*g/kg and 2.125 ± 0.61 *μ*g/kg, respectively, for gboma and dawadawa and were found to be below regulatory limits for both Ghana Standards Authority (10 *μ*g/kg) and the European Food Safety Authority (4 *μ*g/kg) [40]. Seemingly not harmful, it is worthy to note that no amount of aflatoxin is considered safe no matter how minute it is in public health. The Ghanaian public health authorities as well as the populace have to monitor ceaselessly to detect and reduce AFB_1_ contamination in various foods and make conscious efforts to suppress to an ALARA (as low as reasonably achievable) level.

The main mycotoxins that contaminate spices are aflatoxins and ochratoxins. Furthermore, aflatoxin is the most common mycotoxin in spices, and aflatoxin contamination in spices has been studied widely by several researches [41–43].

AFB_1_ was found in 8 of the 10 nutmeg samples tested in Portugal [44], with levels ranging from 1.25 to 58 g/kg, whereas AFB_1_ was found in only one of the three nutmeg products in Italy [6, 44] which disagreed with results of this study as mycotoxins in nutmeg and aniseed were below detection limits. In five nutmeg samples from Egypt, El-Kady et al. [45] found no aflatoxins. Ochratoxins A (OTA) was also not found in nutmeg purchased in Belgium, the Netherlands, or Russia from retail stores [46]. Yarru et al. [47] found that curcumin has partial protective effects on alterations in antioxidant biotransformation and immune system gene expression in the livers of chicks fed AFB_1_. Turmeric powder recorded values 100-200 mg kg^−1^ bw were found to protect mice from AFB_1_ (2 g kg^−1^ bw)-induced toxicity by regulating lipid peroxidation and enhancing the defense system in a comparable investigation [48]. Another study contrasted with Garcia et al. [49] who did similar work on various fungal and bacteria species with different samples recorded fungal contamination and its negative effect on mankind, ocimum, clove, and turmeric aflatoxin analysis, they were below the limit of quantification (LOQ = 2 *μ*g/kg). However, there were little or no detections of aflatoxin in the samples from Ho except for dawadawa. Several studies have been done concerning these spices and others in different countries like India and Portugal, where aflatoxins were detected, which implies that we made a careful move on how to handle our spices since mostly contamination occurs right from poor agricultural, manufacturing, and storage processes. Azzoune et al. [1] reported that of the 36 spice samples analyzed, 23 (63.9%) were contaminated with AFB_1_ at concentrations ranging from 0.2 to 26.50 *μ*g/kg. High levels of AFB_1_ (26.50, 24.34, 19.07, and 14.65 *μ*g/kg) were found in saffron and sweet cumin, respectively. These levels of AFB_1_ are lower than the maximum limits set by Ghanaian regulations (10 *μ*g/kg). Although the residue limit of aflatoxin levels in spices is not well established in Ghana, a limit of 10 *μ*g/kg has been proposed by Ghana Standards Authority [40]. In the European Union, aflatoxin levels in several spices are regulated to maximum residue levels that cannot be greater than 5 *μ*g/kg for AFB_1_ and 10 *μ*g/kg for aflatoxins (AFs) (Commission Regulation (EC) No. 472/2002).

In black pepper, caraway, cinnamon, coriander, cumin, ginger, red pepper, and sweet pepper, AFB_1_ was detected with levels ranging from 0.10 to 3.44 *μ*g/kg, lower than the limit as recognized in Ghana [50]. The occurrence of mycotoxins in spices differs geographically and depending on the climatic conditions. The presence of AFB_1_ in widely varying amounts in spices has been reported by many authors [43, 51, 52]. In agreement with our findings in Ghana, Ahene et al. [9] reported that aflatoxin analysis conducted on the set of spices investigated showed that aflatoxins B_1_, B_2_, G_1_, and G_2_ were not formed at all.

Toxicogenic species are fungal species that produce potentially harmful natural toxic compounds known as mycotoxins [53, 54]. These cause health hazards and even death in humans and animals when they occur in large quantities in food. It is worthy to note that regarding the mycotoxins produced by toxicogenic species, no amount of toxin above the zero level is regarded as safe. “Reduction to as low as reasonably achievable” is the endorsement of JECFA concerning the safe level in foods following the significant genotoxic, carcinogenic, etc. probabilities of these toxins [53].

Results from the present study confirmed the presence of *Aspergillus* species as well as a natural occurrence of aflatoxins. Nonetheless, there is a high probability of the occurrence of diverse types of mycotoxins produced by this species of fungi in the spice and herb samples. *Aspergillus* (*niger*, *parasiticus*, *fumigatus*, *terreus*, and *ochraceus*) species isolated from these spices and herbs in this work confirmed the contamination of aflatoxins (produced chiefly by *A. parasiticus*, *A. flavus*) which is categorized as a class 1 carcinogen [55]. When ingested even at the tiniest quantities via the skin, aflatoxins have teratogenic, carcinogenic, hepatotoxic, and mutagenic outcomes on human health [56], due to their accumulative potential. Persistent intake of foods contaminated with aflatoxins leads to the unadorned and unimaginable conditions listed herein. When food contaminated with aflatoxins is ingested, the aflatoxins are then transformed into aflatoxin-8,9-epoxide metabolites in the liver, which has been implicated in numerous unsafe consequences in the body [57, 58]. Epidemiological studies of human populations exposed to diets naturally contaminated with aflatoxins revealed a connection between the high incidence of liver cancer in Africa and elsewhere and dietary intake of aflatoxins [59]. Aflatoxin assimilation surges the risk of liver cancer by exceeding tenfold compared to either exposure unaccompanied [60].

### 4.4. Antimicrobial Activity

The antibiotic used as a control against the sample extracts exhibited effectiveness against the bacteria. There was a relatively magnificent antimicrobial sensitivity of *Streptococcus mutans* to dawadawa 500 mg/ml as it cleared a zone of 23.50 ± 0.71 mm being the highest inhibition zone recorded. Ginger is well known for its excellent antioxidant and phytochemical composition. In a study by Hasan et al. [61] investigating the effects of different drying methods of ginger on its nutritional composition and antimicrobial properties, ginger produced relatively similar antimicrobial properties with *Salmonella* spp., *S. aureus*, and *Staphylococcus epidermis*. According to the findings, ginger contains monoterpenoids, sesquiterpenoids, phenolic compounds, and their derivatives, as well as aldehydes, ketones, alcohols, and esters, all of which have a broad antibacterial spectrum against various microbes [62]. In another study, Chand [63] reported the effectiveness of ginger against *E. coli*, *Salmonella* sp., *Staphylococci*, and *Streptococci*. Ginger has been reported to be effective on the control of many microorganisms including *L. monocytogenes* [64–66], *Escherichia coli* [65], *Salmonella enteritidis* [67], *Aspergillus niger* [68, 69], and varied fungal species [70–72].

Dawadawa illustrated a fair antimicrobial sensitivity against *Pseudomonas aeruginosa*, *Staphylococcus aureus*, and *Streptococcus mutans* at 500 mg/ml and 250 mg/ml concentrations only, but revealed no antimicrobial potential against *Salmonella typhi* and *Klebsiella pneumoniae* for all concentrations. Olukunle et al. [73] also found that effluent of West African locust beans (*P. biglobosa*) without its chaff inhibited growth on *S. aureus*, *S. typhi*, *E. coli*, and *S. pyogenes*, but *P. aeruginosa* and *K. pneumoniae* were resistant to the treatment. The antimicrobial activity of *P. biglobosa* bark extract was investigated against some microbial isolates. The extract at a concentration of 20 mg/ml was found to inhibit the growth of all fifteen bacterial isolates comprising both Gram-positive and Gram-negative organisms. The zones of inhibition exhibited by the extract ranged between 14 ± 0.00 mm for *E. coli* and 28 ± 0.71 mm for *P. aeruginosa*. The antibacterial properties of alkaloids from *P. biglobosa* have been reported to have the ability to intercalate with DNA [74]. Cardiac glycosides are an important class of naturally occurring drugs whose actions help in the treatment of congestive health failure [75]. This class of phytochemical compounds was detected in *P. biglobosa* extract and thus supports the usefulness of this plant for the treatment of cardiac infections along with other *P. biglobosa* to treat ailments such as dental caries and cough among the Yoruba tribe of southwestern Nigeria because of its antimicrobial activity [75].

Although scanty data exists on the antimicrobial properties of *S. macrocarpon* leaves, the ethanolic extract has been reported to demonstrate increased antimicrobial sensitivity against *S. aureus* from 50 g/100 ml through 75 g/100 ml, 100 g/100 ml, to 150 g/100 ml; their study confirmed the antimicrobial potential of methanolic extract of Gboma leaves against *S. aureus* [76, 77]. Results from this study were in agreement with our findings. Additionally, leaf extracts of some species of *Solanum* spp. have been reported to be effective against some fungal species such as *Aspergillus flavus* [78] and *Penicillium notatum* [78, 79] and bacteria species *Staphylococci* and *Klebsiella pneumoniae* [79].

*Laurus nobilis* L. (bay leaf) is also reported to be effective against several microorganisms. Xu et al. [80] and Peixoto et al. [81] found it to be effective against *Alternaria alternate* and *E.coli* as well as *Candida* spp., respectively. Again, Fidan et al. [82] reported its effectiveness against 4 Gram-positive bacteria and 12 Gram-negative bacteria. Furthermore, Ramos et al. [83] reported its antimicrobial activity against *B. thermosphacta*, *E. coli*, *L. innocua*, *L. monocytogenes*, *P. putida*, *S. typhimurium*, and *Shewanella putrefaciens*. Ceyhan et al. [84] confirmed an inhibitory strength against *B. cereus*, *S. aureus*, *E. coli*, *K. pneumoniae*, and *C. albicans.*

The variation of the rate of resistance can be related to the difference in time and place. Another reason for the difference in resistance rates might be a rapid change in antibiotic sensitivity patterns of bacteria within a short period [85]. The prevalence of antibiotic-resistant bacteria is growing due to the random use of antibiotics in human therapy, animal farming, and other prophylactic usages [86]. Resistance enables bacteria to escape from being killed by antibiotics and reduces the ability to treat infections [87]. Therefore, antibiotic resistance has been considered one of the greatest threats to medicine [87, 88]. The growing concern about food safety has recently led to the development of natural antimicrobials to control foodborne pathogens and spoilage bacteria. Many of the spices and herbs used today have been valued for their antibacterial effects and medicinal powers in addition to their flavor and fragrance qualities [89].

### 4.5. pH of Samples

According to data recorded by the Scientific Sper and the Centre for Food Safety and Applied Nutrition, the pH range of ginger root is 5.60 and an upper pH value of 5.90 (cabbage, 6.00; broccoli, 6.41 when cooked and 6.58 in its raw frozen state) [90]. Most plant-based foods, except for a few, have pH values from 3 to 6, being acidic to slightly acidic. All samples tested in this study had a slightly acidic pH between 5.80 ± 0.00 and 6.76 ± 0.35. Recently, Nieva et al. [91] highlighted that vegetables with slightly acidic pH tend to have a longer shelf life as this property provides microbe- and spoilage-resistant nature.

## 5. Conclusion

From our study, it can be surmised that the local herbs and spices ginger, bay leaf, gboma, and dawadawa harbored a total of 12 species belonging to 7 genera; *Aspergillus* (*niger*, *flavus*, *fumigatus*, and *ochraceus)*, *Fusarium* (*oxysporum*, *verticillioides*), *Mucor* (*racemosus*), *Penicillium* (*digitatum*, *expansum*), *Rhizopus* (*stolonifer*), *Rhodotorula* sp., and *Trichoderma harzianum* were identified as fungal contaminants. *Fusarium oxysporum* was the most predominant species identified. Mycobiota encountered with mycotoxigenic potential and human health importance were *A. niger*, *A. flavus*, *A. fumigatus*, *F. verticillioides*, and *Penicillium verrucosum*. The presence of some of these toxigenic fungi and the subsequent detection of aflatoxins in these herbs and species have the potential of causing adverse health effects, especially hepatocellular carcinoma on the consumer and supply chain populace of the Ho municipality. These herbs and spices investigated also showed sufficient effectiveness against many of the tested microorganisms. Their pH also played an important role in the multiplication of molds of spices and herbs.

Although aflatoxin levels recorded were in small quantities in dawadawa and gboma, it is noteworthy that no minute amount is considered safe in public health since they can accumulate and cause some adverse effects for consumers. Therefore, for the contamination levels to reduce, marginally intensive public awareness should be carried out by FDA on food hygiene practices for local market retailers and food vendors in the local markets for farmers to indulge in good agricultural practices, and proper storage conditions should also be established in order not to expose spices and some herbs to mold infection.

## Figures and Tables

**Table 1:** Spices and herb samples and their corresponding interpretations.

Code	Interpretation
GP1	Ginger powder/vendor 1
GP2	Ginger powder/vendor 2
GP3	Ginger powder/vendor 3
GF1	Ginger fresh/vendor 1
GF2	Ginger fresh/vendor 2
GF3	Ginger fresh/vendor 3
BL1	Bay leaf/vendor 1
BL2	Bay leaf/vendor 2
BL3	Bay leaf/vendor 3
GB1	Gboma/vendor 1
GB2	Gboma/vendor 2
GB3	Gboma/vendor 3
DAW1	Bambara bean/vendor 1
DAW2	Bambara beans/vendor 2
DAW3	Bambara beans/vendor 3

**Table 2:** Percentage occurrence (%) per sample of different fungal species identified. TPO_fs_ = total percentage occurrence of each fungal species among all samples.

Fungal species	Percentage occurrence (%)															TPO_fs_ 100%
	GP1	GP2	GP3	GF1	GF2	GF3	BL1	BL2	BL3	GB1	GB2	GB3	DAW1	DAW2	DAW3	
*Aspergillus niger*	—	55.0	—	15.0	—	7.0	25.0	—	42.0	56.0	33.0	—	5.3	—	5.3	16.24
*Aspergillus flavus*	—	—	—	24.0	3.3	—	—	—	20.0	—	13.8	—	—	—	—	4.07
*Aspergillus fumigatus*	—	—	70.0	17.0	—	—	15.0	—	—	29.0	—	—	5.4	—	5.5	9.46
*Aspergillus ochraceus*	—	15.5	—	—	—	—	—	—	8.0	—	13.9	—	8.9	—	—	3.09
*Fusarium oxysporum*	100.0	29.5	30.0	—	25.3	—	45.0	—	16.0	15.0	35.0	—	25.0	16.7	16.1	23.54
*Fusarium verticillioides*	—	—	—	12.0	—	71.0	—	—	—	—	—	27.0	—	—	—	7.33
*Mucor racemosus*	—	—	—	—	—	—	—	40.0	11.0	—	—	—	—	—	3.4	3.63
*Penicillium digitatum*	—	—	—	—	6.0	22.0	15.0	—	—	—	—	—	55.3	—	—	6.55
*Penicillium expansum*	—	—	—	—	—	—	—	—	—	—	—	73.0	—	—	—	4.90
*Rhizopus stolonifer*	—	—	—	—	65.4	—	—	60.0	—	—	—	—	—	56.7	46.5	15.18
*Rhodotorula* sp.	—	—	—	21.0	—	—	—	—	—	—	4.3	—	—	—	3.8	1.94
*Trichoderma harzianum*	—	—	—	11.0	—	—	—	—	3.0	—	—	—	—	26.6	19.4	4.00
Total no. of species	1	3	2	6	4	3	4	2	6	3	5	2	5	3	7	

**Table 3:** Pooled data of total fungi isolated from ginger, dawadawa, bay leaf, and gboma.

*Aspergillus niger* Van Tieghem^GP2,GF1,GF3,BL1,BL3,GB1,GB2,DAW1,DAW3^
*A. flavus* Link^GF1,GF2,BL3,GB2^
*A. fumigatus* Fresen^GP3,GF1,BL1,GB1,DAW1^
*A. ochraceus* Wilhelm^GP2,BL3,GB2,DAW1^
*Fusarium oxysporum* Schlecht^GP1,GP2,GP3,GF2,BL1,BL3,GB1,GB2,DAW1,DAW2,DAW3^
*Fusarium verticillioides* ^GF1,GF3,GB3^
*Mucor racemosus* Fres.^BL2,BL3,DAW3^
*Penicillium digitatum* Sacc.^GF3,BL1^
*Penicillium expansum* Dierckx^GB3^
*Rhizopus stolonifer* (Ehrenb.) Lind.^GF2,BL2,DAW2,DAW3^
*Rhodotorula mucilaginosa* (A. Jorg) F.C. Harrison^GF1,GB2,DAW3^
*Trichoderma harzianum* Rifai^GF1,BL3,DAW2,DAW3^

**Table 4:** Pooled data of antimicrobial activities of spices and herbs against microorganisms.

Samples	Concentration (mg/ml)	Zone of inhibition (mean in mm ± std)				
		PA	KP	SA	SM	ST
Ginger	500	4.50 ± 6.36	NZI	11.50 ± 2.12	10.50 ± 0.71	NZI
	250	8.00 ± 0.00	9.50 ± 2.12	11.50 ± 3.54	8.50 ± 0.71	7.00 ± 0.00
	125	9.50 ± 0.70	9.00 ± 0.00	12.00 ± 5.66	10.00 ± 0.00	11.00 ± 2.83
	62.5	7.50 ± 0.70	10.00 ± 2.82	10.50 ± 3.54	8.50 ± 0.71	8.50 ± 0.71
	Ctrl	13.50 ± 0.70	29.00 ± 1.41	24.00 ± 5.66	19.00 ± 1.41	34.00 ± 7.00

Bay leaves	500	9.00 ± 0.00	NZI	10.00 ± 0.00	9.00 ± 2.82	NZI
	250	7.00 ± 0.00	NZI	7.50 ± 0.71	9.00 ± 2.82	NZI
	125	6.00 ± 0.00	NZI	6.00 ± 0.00	9.00 ± 4.24	NZI
	62.5	6.00 ± 0.00	NZI	6.00 ± 0.00	NZI	NZI
	Ctrl	20.00 ± 7.07	28.50 ± 0.71	26.50 ± 0.71	29.50 ± 0.71	33.00 ± 0.00

Gboma	500	13.00 ± 1.41	9.50 ± 0.71	8.50 ± 2.12	11.00 ± 0.00	NZI
	250	8.00 ± 1.41	8.00 ± 1.41	7.50 ± 0.71	7.00 ± 0.00	NZI
	125	7.00 ± 0.00	8.00 ± 0.00	7.50 ± 0.71	NZI	NZI
	62.5	7.50 ± 0.71	NZI	NZI	NZI	NZI
	Ctrl	10.50 ± 0.71	29.00 ± 1.41	25.00 ± 0.00	15.00 ± 0.00	NZI

Dawadawa	500	15.00 ± 0.00	NZI	16.50 ± 0.71	23.50 ± 0.71	NZI
	250	10.00 ± 0.00	NZI	9.50 ± 0.71	14.00 ± 0.00	NZI
	125	NZI	NZI	NZI	NZI	NZI
	62.5	NZI	NZI	NZI	NZI	NZI
	Ctrl	22.50 ± 3.54	33.5 ± 4.95	25.00 ± 0.00	15.00 ± 0.00	27.5.0 ± 3.54

Mean zone of inhibition of extracts from four spices and herbs against bacterial species. PA = *Pseudomonas aeruginosa*; KP = *Klebsiella pneumoniae*; SA = *Staphylococcus aureus*; SM = *Streptococcus mutans*; ST = *Salmonella typhi*; NZI = no zone of inhibition; control (Ctrl) = chloramphenicol 30 *μ*g.

**Table 5:** pH of spices and herbs from the Ho municipality.

Sample	Mean ± std
Ginger powder	6.56 ± 0.01^b^
Fresh ginger	6.50 ± 0.00^b^
Gboma	5.81 ± 0.00^a^
Bay leaf	6.20 ± 0.01^b^
Dawadawa	6.76 ± 0.04^b^

Means with the same superscripts in a column are not significantly different (*p* > 0.05).

## Data Availability

The datasets used during the current study are available from the corresponding author on request.
